# Yu Shi An Chang Fang Ameliorates TNBS-Induced Colitis in Mice by Reducing Inflammatory Response and Protecting the Intestinal Mucosal Barrier

**DOI:** 10.1155/2021/8870901

**Published:** 2021-05-04

**Authors:** Bin Ye, Liqun Lai

**Affiliations:** Digestive Department, Hangzhou TCM Hospital, Hangzhou 310000, Zhejiang, China

## Abstract

Ulcerative colitis (UC) is an inflammatory bowel disease that is related to the occurrence of colon cancer. This study aimed to investigate the underlying mechanism by which Yu Shi An Chang Fang (YST) treated UC. 2, 4, 6-trinitrobenzene sulfonic acid (TNBS) was used to construct the UC model. The body weight, fecal viscosity, and fecal bleeding of all mice were recorded every day to calculate the DAI value. The pathological changes in colon tissues were observed by hematoxylin-eosin (H&E) staining. The levels of tumor necrosis factor-*α* (TNF-*α*), interleukin-1 beta (IL-1*β*), interleukin-6 (IL-6), and myeloperoxidase (MPO) reflecting inflammation and the levels of malondialdehyde (MDA), glutathione peroxidase (GSH-Px), and superoxide dismutase (SOD) reflecting oxidative stress in colon tissues were all measured by their assay kits. The mRNA expression of TNF-*α*, IL-1*β,* and IL-6 in colon tissues was detected by quantitative reverse transcription-PCR (qRT-PCR). The expression of proteins related to pyroptosis and the colonic mucosal barrier was analyzed by Western blot. As a result, TNBS caused decreases in body weight and colon lengths, triggered serious histological damage, promoted inflammation, oxidative stress, and pyroptosis, and destroyed the colonic mucosal barrier. The above changes caused by TNBS in colitis mice could be partially reversed by YST. In conclusion, YST ameliorates TNBS-induced UC in mice by reducing the inflammatory response and protecting the intestinal mucosal barrier.

## 1. Introduction

Ulcerative colitis (UC), also known as chronic nonspecific ulcerative colitis, is characterized by inflammation and ulcerative lesions in the rectal (sigmoid) colonic mucosa and submucosa, and the entire colon and terminal ileum can be destroyed in severe cases [1]. UC is difficult to cure and has a high recurrence rate, which is associated with the occurrence of colon cancer [2]. Listed as one of the troublesome diseases by the World Health Organization, this disease has been widely valued by the medical community. According to a report, the incidence of UC in North America and Europe is as high as 23.14/100,000 and 57.9/100,000, respectively [3]. The latest epidemiological data show that the prevalence and incidence of UC in the Chinese mainland are on the rise sharply, with incidence 1.33/100,000 at present [4].

Important pathogenesis of UC is the imbalance of proinflammatory cytokines and anti-inflammatory cytokines, which means the content of serum anti-inflammatory factors in the UC active period is lower than that in the remission period, while the content of proinflammatory factors is higher than that in the remission period [5]. Moreover, the serum levels of tumor necrosis factor-*α* (TNF-*α*), interleukin-6 (IL-6), and interleukin-8 (IL-8) in UC patients are higher than those in the control group. There are significant differences in serum levels of TNF-*α*, IL-6, and IL-8 in patients with UC at different stages [6]. Cytokine imbalance in the human body immediately leads to the activation of various inflammatory cells to release a variety of inflammatory factors, which ultimately induces a chronic inflammatory response in the intestinal tissues. In addition, the colonic mucosal barrier plays an important role in the pathogenesis of UC [7, 8]. A recent study has shown that intestinal epithelial cells are a highly dynamic tissue that can respond to signals transmitted by microbiota and immune system, thereby regulating barrier function. Meanwhile, abnormal signal transduction between epithelial cells and neighboring immune cells may promote immune dysregulation of UC [9]. The colonic mucosal barrier includes epithelial cells and the intercellular tight junction (TJ) [10, 11]. The disruption of TJ can destroy the paracellular barrier, thus leading to the enhancement in mucosal permeability and drastic mucosal inflammation in UC [12, 13].

Yu Shi An Chang Fang (YST) is a typical Chinese medicine prescription for treating UC rats through alleviating the occurrence of mucosal hyperplasia and ulcer and improves the level of inflammatory factors such as TNF-*α*, IL-8, and IL-10 in serum and intestinal mucosa [14]. The main ingredients of YST are *Scutellaria baicalensis* 20 g, *Astragalus* 15 g, *Atractylodes* 10 g, *Licorice* 10 g, *Radix Paeoniae Alba* 20 g, *Cinnamon* 5 g, *Pueraria* 15 g, *Carbonized Catnip* 15 g, *Radix Sanguisorbae* 30 g, *Bletilla Striata* 10 g, *Frankincense* 5 g, *Herba Patriniae* 30 g, *Sargent Gloryvine* 15 g, and *Panax Notoginseng powder* 3 g. However, there is a lack of research related to the underlying mechanism by which YST treats UC.

Therefore, the purpose of this study was to investigate the mechanism by which YST alleviates 2, 4, 6-trinitrobenzene sulfonic acid (TNBS) induced UC in mice.

## 2. Materials and Methods

### 2.1. Colitis Mice and YST Treatment

The program was approved by the Animal Experimental Ethics Review Committee of Hangzhou TCM Hospital. Twenty male BALB/c mice (8 weeks, 17–20 g) were purchased from the Animal Experimental Center of Zhejiang Province. All mice were randomly divided into four groups, named as the control group (Control, 0.9% saline), colitis group (Model, 100 mg/kg TNBS), low-YST dose group (YST-L, 1 mL/kg YST), and high-YST dose group (YST-H, 10 mL/kg YST). To induce colitis, 5 mg of TNBS was dissolved in 0.2 ml of 50% ethanol. Each group consisted of five mice.

Each group except the control group was modeled. After anesthesia, mice were given 100 mg/kg TNBS every day by rectal perfusion. After seven days, mice in YST-L group and YST-H group were, respectively, given 1 mL/kg YST and 10 mL/kg YST by gavage every day, lasting 7 days. A total of 3 mL of YST solution and normal saline was given by gavage each time. The control group and the model group were given 3 mL normal saline by gavage. At the end of the experiment, all mice were fasted for 24 h and euthanized. There was no death of mice during the model construction. Colon lengths were recorded and colon specimens were divided into two sections. One section was frozen in liquid nitrogen for further analysis, and the other was immediately fixed in 4% paraformaldehyde solution, prepared by conventional methods after 48 h. After hematoxylin-eosin (H&E) staining, pathological changes of the colon were observed under an inverted microscope.

### 2.2. Disease Activity Index (DAI)

At the beginning of the experiment, the DAI of all the mice was evaluated daily. DAI was a comprehensive score of weight loss, fecal viscosity, and fecal bleeding [15]. DAI = (weight loss score + stool character score + stool blood condition score)/3.

### 2.3. Enzyme-Linked Immunosorbent Assay (ELISA)

Colon tissues from all mice were homogenized with lysis buffer and centrifuged at 1000 r/min at 4°C for 15 min to obtain the supernatant. The levels of tumor necrosis factor-*α* (TNF-*α*) (cat. no. PT512), interleukin-1 beta (IL-1*β*) (cat. no. PI301), and interleukin-6 (IL-6) (cat. no. PI326) in the colon supernatant were determined by ELISA kits (Beyotime) according to the manufacturer's protocol.

### 2.4. Quantitative Reverse Transcription-PCR (qRT-PCR)

The colon tissues were fully ground to powder in liquid nitrogen. After grinding, Trizol reagent was added to EP tube, which was then centrifuged to obtain the total RNAs. The cDNAs of TNF-*α*, IL-1*β,* and IL-6 were synthesized using the Transcriptor First Strand cDNA Synthesis Kit and qPCR analysis conducted by Power SYBR Green. The thermocycling conditions were as follows: initial denaturation at 95°C for 5 min, followed by 35 cycles of denaturation at 95°C for 15 s, annealing at 60˚C for 30 s, and extension at 72°C for 25 s. The primer sequences were as follows: TNF-*α* forward, 5′-CAA AGT AGA CCT GCC CAG AC-3′; reverse, 5′-GAC CTC TCT CTA ATC AGC CC-3′; IL-1*β* forward, 5′-AAA AGC TTG GTG ATG TCT GG-3′; reverse, 5′-TTT CAA CAC GCA GGA CAG G-3′; IL-6 forward, 5′-GTG TGA AAG CAG CAA AGA GGC-3′; reverse, 5′-CTG GAG GTA CTC TAG GTA TAC-3′; GAPDH forward, 5′-CATGAGAAGTATGACAACAGCCT-3′ and reverse, 5′-AGTCCTTCCACGATACCAAAGT-3′. The mRNA expression of TNF-*α*, IL-1*β,* and IL-6 was calculated by the 2^−△△CT^ method with the internal reference as GAPDH.

### 2.5. Western Blot Analysis

After extraction of proteins from colon tissues, the determination of protein concentration was dependent on the BCA Protein Assay kit. 30 *µ*g sample per lane was uploaded and separated through 12% SDS-PAGE gradient gel. The proteins were transferred to the PVDF membrane, which was sealed with 5% nonfat milk at 4°C for 2 h and added with primary antibodies including pyrin domain containing 3 (NLRP3) (ab263899, Abcam), anti-ASC (cat. no. AG-25B-0006, Adiogen), anti-pro caspase-1 (ab52079, Abcam), anti-Cleaved caspase-1 (#89332, Cell signaling pathway), anti-ZO1 (ab276131, Abcam), anti-Occludin (OCLN) (ab216327, Abcam), and anti-Claudin 1 (CLDN1) (ab180158, Abcam) at 4°C overnight. The next day, the membrane was incubated with the appropriate antihorseradish peroxidase-conjugated antibody and visualized through enhanced chemiluminescence detection. The gray value of the target protein expression was calculated by Image-Pro Plus software.

### 2.6. Measurement of Myeloperoxidase (MPO) Activity and Oxidative Stress Level

MPO activity was used to reflect whether neutrophil infiltration into inflamed colonic mucosa happened. The MPO activity (ab155458, Abcam) was measured by the O-dianisidine method, and levels of malondialdehyde (MDA) (cat. no. S0131S, Beyotime), glutathione peroxidase (GSH-Px) (cat. no. S0056, Beyotime), and superoxide dismutase (SOD) (cat.no. S0103, Beyotime) were quantified by their assay kits in the supernatant of colonic tissues.

### 2.7. Statistical Analysis

All the data presented as mean ± standard deviation were analyzed with the SPSS 22.0. One-way analysis of variance (ANOVA) was used to compare the significance of the differences in multiple groups, followed by Tukey's test. *P* < 0.05 was considered a significant difference among groups.

## 3. Results

### 3.1. Effects of YST on Clinical Manifestations of TNBS-Induced Colitis Mice

**Figure 1:**
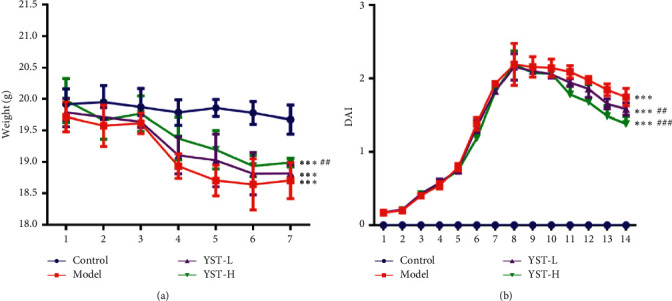
Effects of YST on clinical manifestations of TNBS-induced colitis mice. (a) Body weight. (b) DAI value. ^*∗∗∗*^*P* < 0.001 vs. Control group. ^##^*P* < 0.01 and ^###^*P* < 0.001 vs. Model group.

The body weights of mice in the Model group were obviously decreased and YST from low dose to high dose gradually improved the body weights of mice (Figure 1(a)). No significant difference was found in DAI values in the Model group, YST-L group, and YST-H group in the first 7 days, and YST from low dose to high dose downregulated the DAI value, especially from day 11 to day 14 (Figure 1(b)).

### 3.2. Effects of YST on Colon Tissues of TNBS-Induced Colitis Mice

**Figure 2:**
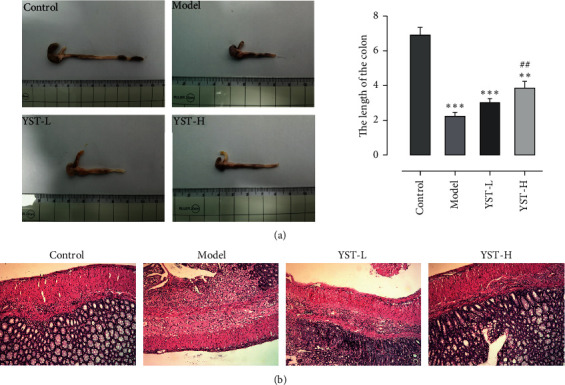
Effects of YST on colon tissues of TNBS-induced colitis mice. (a) Representative picture of the colon and colon length. (b) Representative image of mouse colon stained with H & E. ^*∗∗*^*P* < 0.01 and ^*∗∗∗*^*P* < 0.001 vs. Control group. ^##^*P* < 0.01 vs. Model group.

The colon length of mice in the Model group became shorter, which was increasingly improved by YST from low dose to high dose. Of note, the colon length of mice in the YST-H group was longer than that in the YST-L group (Figure 2(a)). TNBS induction resulted in colonic mucosa injury and inflammatory cell infiltration that were significantly ameliorated by YST therapy (Figure 2(b)).

### 3.3. YST Alleviates Inflammation and Oxidative Stress in TNBS-Induced Colitis Mice

**Figure 3:**
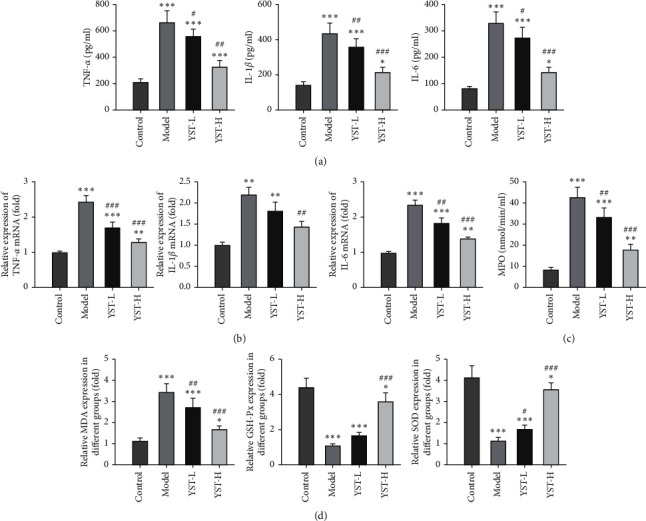
YST alleviates inflammation and oxidative stress in TNBS-induced colitis mice. (a) The levels of TNF-*α*, IL-1*β,* and IL-6 in the colon tissue. (b) The mRNA expression of TNF-*α*, IL-1*β,* and IL-6 in the colon tissue. (c) MPO activity in the colon tissue. (d) MDA, GSH-Px, and SOD concentrations in the colon tissue. ^*∗*^*P* < 0.05, ^*∗∗*^*P* < 0.01 and ^*∗∗∗*^*P* < 0.001 vs. Control group. ^#^*P* < 0.05, ^##^*P* < 0.01 and ^###^*P* < 0.001 vs. Model group.

Inflammation and oxidative stress are classic features of colitis, and thus the inflammatory cytokines and factors related to oxidative stress were detected herein for the verification of the role of YST in the TNBS-induced colitis mice model. The levels of TNF-*α*, IL-1*β,* and IL-6 in colon tissues of colitis mice were elevated by contrast with those in the Model group. YST from low dose to high dose gradually decreased the levels of TNF-*α*, IL-1*β,* and IL-6 in colon tissues of colitis mice (Figure 3(a)). The changes in mRNA expression of TNF-*α*, IL-1*β,* and IL-6 in these four groups were similar to those in levels of TNF-*α*, IL-1*β,* and IL-6 (Figure 3(b)). As shown in Figure 3(c), MPO activity was enhanced in colon tissues of colitis mice, while YST suppressed the MPO activity (Figure 3(c)). MDA expression was decreased and the expression of GSH-Px and SOD was increased in colon tissues of colitis mice, which was reversed by YST treatment (Figure 3(d)).

### 3.4. YST Improves TNBS-Induced Colitis by Inhibiting Pyroptosis

**Figure 4:**
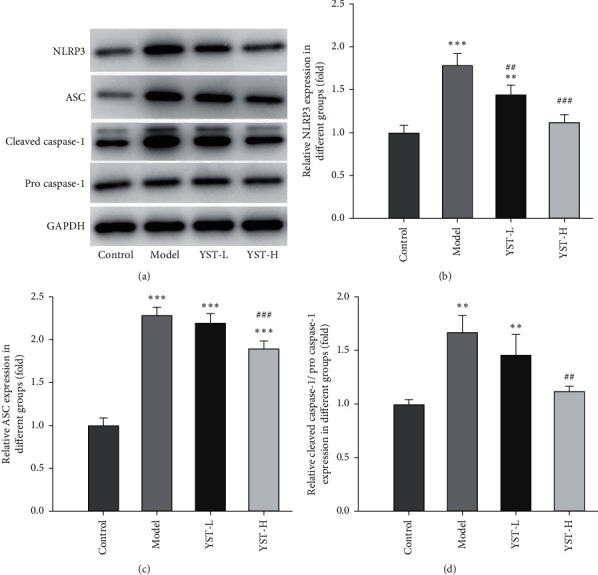
YST improves TNBS-induced colitis by inhibiting pyroptosis. The expression of NLRP3, ASC, Cleaved caspase-1, and Pro-caspase-1 in the colon tissue was detected by Western blot analysis. ^*∗∗*^*P* < 0.01 and ^*∗∗∗*^*P* < 0.001 vs. Control group. ^##^*P* < 0.01 and ^###^*P* < 0.001 vs. Model group.

The expression of pyroptosis related proteins (NLRP3, ASC, and Cleaved caspase-1) was upregulated in colon tissues of colitis mice and YST downregulated the above protein expression (Figure 4).

### 3.5. YST Improves TNBS-Induced Colitis by Protecting Integrity of the Colonic Mucosal Barrier

**Figure 5:**
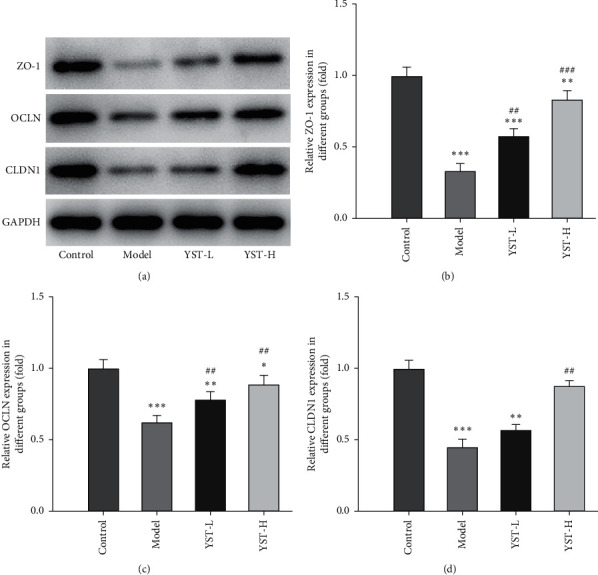
YST improves TNBS-induced colitis by protecting the integrity of the colonic mucosal barrier. The expression of ZO-1, OCLN, and CLDN1 in the colon tissue was detected by Western blot analysis. ^*∗*^*P* < 0.01, ^*∗∗*^*P* < 0.01 and ^*∗∗∗*^*P* < 0.001 vs. Control group. ^##^*P* < 0.01 and ^###^*P* < 0.001 vs. Model group.

Structural impairment of the colonic mucus barrier is a critical factor for the pathogenesis of colitis at the early stage [16]. The decreased expression of tight junction proteins (ZO-1, OCLN, and CLDN1) in colon tissues of colitis mice challenged by TNBS was enhanced by YST treatment, indicating the defective colonic mucosal barrier could be restored by YST (Figure 5).

## 4. Discussion

TNBS can destroy the integrity of the colonic mucosal barrier, increase epithelial permeability, and induce strong inflammatory reaction, which enables the use of TNBS to be a typical method to construct the UC animal model [17, 18]. In this study, TNBS induction caused decreases in body weights and colon lengths and serious histological damage in colitis mice, which was similar to the results presented in previous studies [19, 20].

Although western medicine can instantly relieve symptoms of patients in previous treatments, the relapse of disease and the toxic and side effects of repeated drugs after treatment, all affect the treatment effect, safety, physical tolerance, and cooperation duration of UC patients. Thanks to minor side effects, Chinese medicine possessing anti-inflammatory effects has been increasingly investigated in recent decades [21–24]. The main ingredients of YST are *Scutellaria baicalensis* 20 g, *Astragalus* 15 g, *Atractylodes* 10 g, *Licorice* 10 g, *Radix Paeoniae Alba* 20 g, *Cinnamon* 5 g, *Pueraria* 15 g, *Carbonized Catnip* 15 g, *Radix Sanguisorbae* 30 g, *Bletilla Striata* 10 g, *Frankincense* 5 g, *Herba Patriniae* 30 g, *Sargent Gloryvine* 15 g, *Panax Notoginseng powder* 3 g. *Scutellaria baicalensis Georgi* ,and *Paeonia lactiflora Pall* could treat UC [25, 26]. *Cinnamaldehyde* (CA) is a main active ingredient of *cinnamon* and CA could alleviate dextran sulfate sodium (DSS)-induced colitis by inhibiting activation of NLRP3 inflammasome [27]. One herb of Qingre Zaoshi Liangxue Fang (QRZSLXF) was *Bletilla Striata* and QRZSLXF could also treat UC by regulating the DOR-*β*-arrestin1-Bcl-2 signal transduction pathway [28]. Inflammation and oxidative stress in various diseases could be alleviated by the ingredients in YST, such as *Scutellaria baicalensis* [29]*, Astragalus* [30]*, Atractylodes* [31]*, Licorice* [32]*, Penta-O-galloyl-β-d-glucose (obtained from Radix Paeoniae Alba)* [33]*, Cinnamon* [34]*, Mangiferin (obtained from Pueraria)* [35]*, Bletilla Striata* [36]*, and Casperome (extracted from Frankincense)* [37]. In this study, we found that YST effectively suppressed TNBS-induced colonic inflammation showed by reversing changes in body weight, DAI, colonic length, and histological damage in the Model group. Moreover, the colonic levels of MPO and levels of TNF-*α*, IL-1*β*, IL-6, and MDA were decreased, and the levels of GSH-Px and SOD were increased in the YST-treated mice, thus demonstrating the anti-inflammatory and antioxidative stress properties of YST.

The integrity of the intestinal barrier depends on the robust innate immune responses, epithelial paracellular permeability, epithelial cell integrity, and the production of mucus [38]. Chitosan could protect against UC in mice by promoting dominant intestinal microflora and regulating the expressions of TNF-*α*, CLDN1, OCLN, and ZO-1 [39]. Artesunate could feasibly relieve people from UC by increasing the expression of intestinal mucosal barrier-related proteins and inhibiting inflammatory response [40]. Huang-lian-Jie-du Decoction (HLJDD) could suppress the NF-*κ*B signaling pathway and enhance intestinal barrier function to treat DSS-induced UC mice [41]. In addition, swelling, plasma membrane lysis, chromatin fragmentation, and the release of proinflammatory substances of cells, which are presentations of pyroptosis, are closely related to the occurrence and development of UC [42–44]. *Curcumin* could significantly inhibit the activation of NLRP3 inflammasome in DSS-induced mice, reduce the expression of IL-1, IL-6, and other inflammatory cytokines, and alleviate the symptoms of colitis in DSS-induced mice [45]. The therapeutic effect of *Baicalin* on DSS-induced UC mice was positively correlated with its dosage, and *Baicalin* inhibited the activation of nuclear transcription factor-*κ*B (NF-*κ*B) and NLRP3 inflammasome, and at the same time reduced the levels of TNF-*α* and IL-1 in serum and colon tissues [46]. Therefore, we speculated that the effect of YST might partially suppress the pyroptosis and enhance the intestinal mucosa tight junctions as the colonic mucosal barrier. In this study, the expression of NLRP3, ASC, and Cleaved caspase-1 was increased while the expression of CLDN1, OCLN, and ZO-1 was decreased in colon tissues of colitis mice, which were reversed by YST to near normal levels.

In conclusion, YST ameliorates TNBS-induced UC in mice by reducing inflammatory response and pyroptosis and improves the intestinal barrier function, uncovering the underlying mechanism of the protective effect of YST on UC.

## Data Availability

Data will be available upon request to the corresponding author.
